# Nobiletin ameliorates high fat-induced disruptions in rhythmic glucagon-like peptide-1 secretion

**DOI:** 10.1038/s41598-022-11223-7

**Published:** 2022-05-04

**Authors:** Alexandre Martchenko, Andrew D. Biancolin, Sarah E. Martchenko, Patricia L. Brubaker

**Affiliations:** 1 Department of Physiology, University of Toronto, Rm 3366 Medical Sciences Building, 1 King’s College Circle, Toronto, ON M5S 1A8 Canada; 2 Department of Medicine, University of Toronto, Toronto, ON Canada

**Keywords:** Physiology, Metabolism, Metabolic diseases, Obesity

## Abstract

The incretin hormone glucagon-like peptide-1 (GLP-1) is secreted by the intestinal L cell in response to nutrient intake. However, GLP-1 secretion also follows a circadian rhythm which is disrupted by the saturated fatty acid palmitate in vitro and high-fat diet (HFD) feeding in vivo. The flavonoid nobiletin is a clock enhancer which improves metabolic homeostasis. Therefore, the aim of this study was to elucidate whether and how nobiletin mitigates the negative effects of palmitate and HFD-feeding on rhythmic GLP-1 release. Pre-treatment of murine GLUTag L cells with palmitate dampened the GLP-1 secretory response at the normal peak of secretion, while nobiletin co-treatment restored GLP-1 secretion and upregulated the ‘metabolic pathway’ transcriptome. Mice fed a HFD also lost their GLP-1 secretory rhythm in association with markedly increased GLP-1 levels and upregulation of L cell transcriptional pathways related to ‘sensing’ and ‘transducing’ cellular stimuli at the normal peak of GLP-1 release. Nobiletin co-administration reduced GLP-1 levels to more physiological levels and upregulated L cell ‘oxidative metabolism’ transcriptional pathways. Furthermore, nobiletin improved colonic microbial 16S rRNA gene diversity and reduced the levels of Proteobacteria in HFD-fed mice. Collectively, this study establishes that nobiletin improves the normal rhythm in GLP-1 secretion following fat-induced disruption.

## Introduction

Circadian rhythms are endogenous biological patterns that follow a period of approximately 24-h^1–3^. At the molecular level, these rhythms are generated by an autoregulatory transcriptional/translational feedback loop, wherein the BMAL1/CLOCK heterodimer acts as the positive arm to stimulate expression of the genes encoding period (PER) and cryptochrome (CRY)^1–3^. The PER/CRY heterodimer serves as the negative arm and feeds back to repress the expression of BMAL1 and CLOCK^1–3^, with additional inputs provided by REV-ERBα, which inhibits the gene encoding BMAL1 (*Arntl*) and RORα, which stimulates *Arntl*. Although the ‘master’ clock is expressed in the suprachiasmatic nuclei, where it is entrained by light, circadian expression of the clock genes has been well established in peripheral metabolic tissues, including the intestine, pancreatic α and β cells, hepatocytes, skeletal muscle, and adipose tissue^4–11^. Furthermore, the composition and function of the gut microbiome, a crucial determinant of metabolic homeostasis, has also been shown to follow a circadian rhythm^12,13^. Circadian rhythmicity in these tissues is mainly entrained by nutrient intake, as determined by the wake/sleep–feeding/fasting cycle, and their coordinated actions throughout the active period are essential for facilitating proper digestion, absorption, and utilization of ingested nutrients^14^. Consistent with the idea that circadian rhythms are important determinants of metabolic processes, epidemiological data have linked circadian disruption, as in individuals who conduct shiftwork, to the development of metabolic diseases such as type 2 diabetes (T2D) and obesity^15^.

The incretin hormone, glucagon-like peptide-1 (GLP-1), is secreted from the intestinal L cell in response to food intake. GLP-1 plays a critical role in the maintenance of metabolic homeostasis, largely through its actions to enhance glucose-stimulated insulin secretion and reduce insulin resistance indirectly by inducing satiety^16^. Interestingly, patients with metabolic dysregulation, as in the case of T2D and obesity, have been shown to exhibit impaired secretion of GLP-1^17,18^. GLP-1 receptor agonists and degradation inhibitors are now, therefore, widely used to reduce glycemia and lower body weight in patients with T2D or obesity^16^.

The intestinal L cell has recently been shown to be under circadian regulation, with GLP-1 secretion in response to identical glucose loads being greater at the onset of the dark/feeding period as compared to the light/fasting period in rodents, and to vary by time-of-day in humans^19–24^. Furthermore, studies in humans, rodents and isolated β-cells have positioned GLP-1 as an important entrainer of the normal insulin circadian rhythm^19,21,25,26^. Interestingly, obesogenic feeding in rodents, a known disruptor of circadian rhythms, increases oral glucose-stimulated GLP-1 levels throughout the 24-h day, but causes a complete loss of the normal rhythm in the GLP-1 secretory response^21,27^. Transcriptomic analysis has additionally revealed a disruption in core clock gene expression in primary L cells isolated from mice fed a high-fat/high-sucrose western diet (WD)^21^. Furthermore, exposure of the well-established murine (m) GLUTag L cell line to the saturated fatty acid palmitate, a major component of obesogenic diets, also results in loss of the GLP-1 secretory rhythm and disrupts clock gene expression, but is not associated with L cell hypersecretion^27,28^. The discrepancy between these in vivo and in vitro phenotypes is thought to be driven by diet-induced changes in the in vivo environment and, specifically, in the gut microbiome^21^, which has been shown to be an important determinant of rhythmic GLP-1 secretion^29^. Furthermore, obese subjects demonstrate loss of the normal GLP-1 secretory rhythm^30^. However, the molecular pathways within L cells that are disrupted by either palmitate or HFD-feeding remain unclear, as do possible approaches to restore the normal GLP-1 secretory patterns.

The flavonoid compound, nobiletin, has recently been identified as a clock-enhancing molecule, acting as a direct RORα agonist^31^. Nobiletin has also been shown to exert protective effects against metabolic dysfunction, improving glucose tolerance and restoring hepatic clock gene oscillations in HFD-fed mice^31,32^. These actions were determined to be clock-dependent as the metabolic effects of nobiletin were lost in *Clock*^*Δ19/Δ19*^ mice^31^. Furthermore, treatment of T2D human islets, which have a disrupted circadian clock, with nobiletin improves circadian gene oscillations and restores glucose-stimulated insulin secretion, albeit some of these effects may be clock-independent^33,34^. As a recent report indicated that olive oil-induced GLP-1 secretion is increased following nobiletin administration^35^, the goal of the present study was to determine the effects of nobiletin on time-dependent GLP-1 secretion and the mechanism(s) by which nobiletin affects L cell function, under both normal and obesogenic, high-fat conditions.

## Results

### Nobiletin restores stimulated GLP-1 secretion in mGLUTag L cells exposed to palmitate

**Figure 1:**
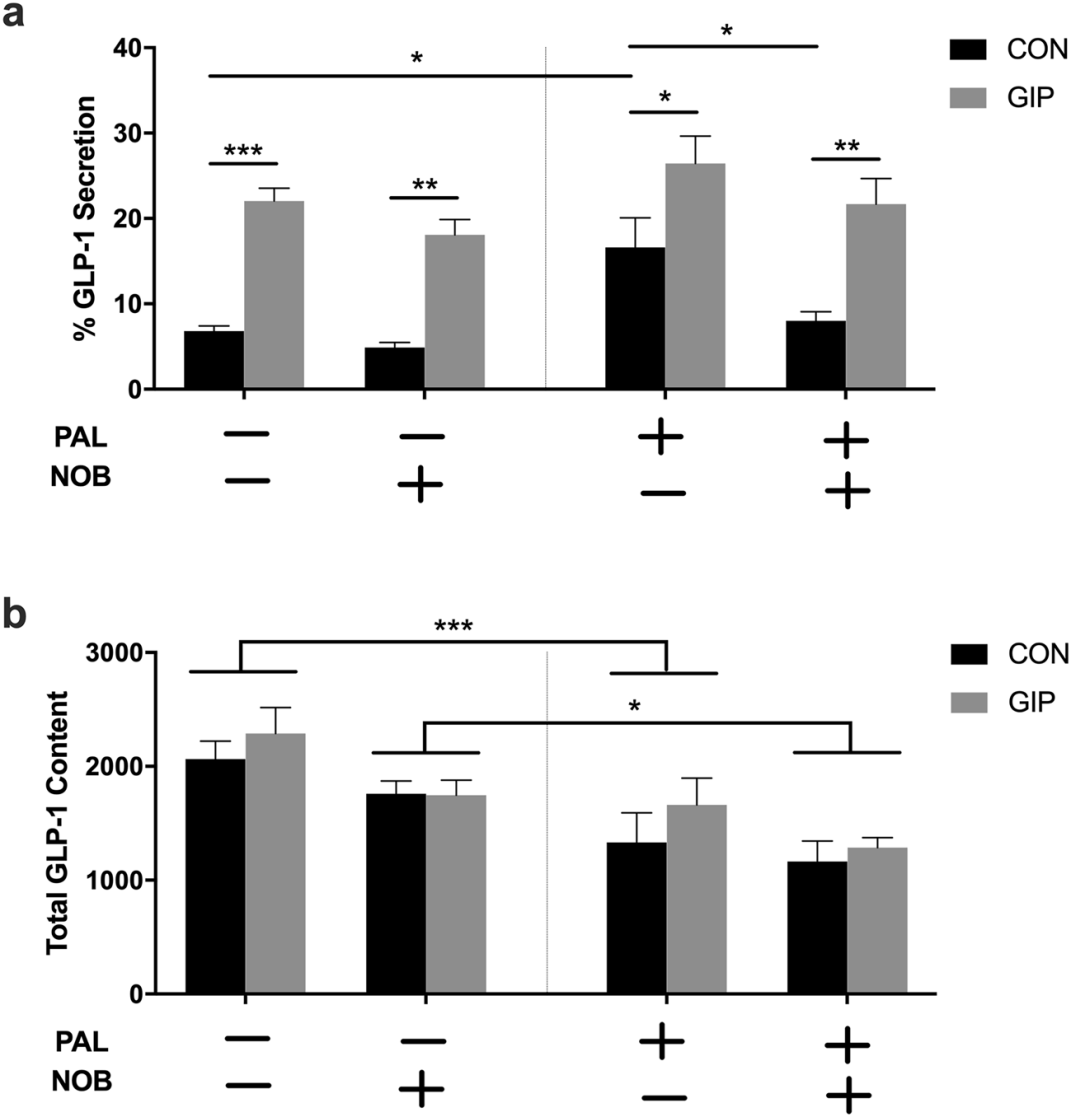
Nobiletin improves the GLP-1 secretory response in mGLUTag L cells following palmitate-induced disruption. (**a**) GLP-1 secretion in response to vehicle (control; CON)- or 10^–7^ M GIP and (**b**) total GLP-1 content, at 8 h following synchronization in mGLUTag L cells pre-treated with a combination of 0.5 mM palmitate (PAL) or vehicle and 20 μM nobiletin (NOB) or vehicle. n = 6; *p < 0.05 **p < 0.01 ***p < 0.001.

To determine the effects of nobiletin on GLP-1 secretion, mGLUTag L cells were pre-treated with and without palmitate and/or nobiletin, and a GIP-induced GLP-1 secretion assay was performed at the established 8-h peak secretory timepoint^19,20,22,28^. As expected, GIP significantly stimulated GLP-1 secretion in normal, vehicle-treated cells (3.2-fold increase; p < 0.001) as well as in palmitate-treated cells (1.6-fold increase; p < 0.05) (Fig. 1a). However, as previously reported^27,28,36^, the fold change in GIP-stimulated GLP-1 secretion was significantly (p < 0.001) reduced in palmitate exposed cells, largely as a result of an increase (p < 0.05) in basal GLP-1 release without a concomitant increase in the secretory response to GIP (Fig. 1a). Furthermore, palmitate significantly (p < 0.001) decreased total GLP-1 content (Fig. 1b), an effect that occurs in the absence of cell death, as previous reported^27,28,36^.

Treatment of normal, vehicle-treated mGLUTag L cells with nobiletin did not alter GLP-1 release with respect to either basal or GIP-induced secretion, and did not affect total GLP-1 content (Fig. 1a,b). However, in the presence of palmitate, nobiletin treatment significantly (p < 0.05) reduced basal GLP-1 secretion, restoring it to the level seen in vehicle-treated cells. Consequently, the normal (2.7-fold increase; p < 0.01) GLP-1 secretory response to GIP was reinstated (Fig. 1a). Nobiletin treatment did not alter the suppressive effect of palmitate (p < 0.05) on total GLP-1 content (Fig. 1b). Taken together, these data indicate that nobiletin restores the normal GLP-1 secretory phenotype in mGLUTag L cells following disruption caused by exposure to the saturated fatty acid palmitate.

### Nobiletin augments ‘metabolic processes’ in mGLUTag L cells exposed to palmitate

**Figure 2:**
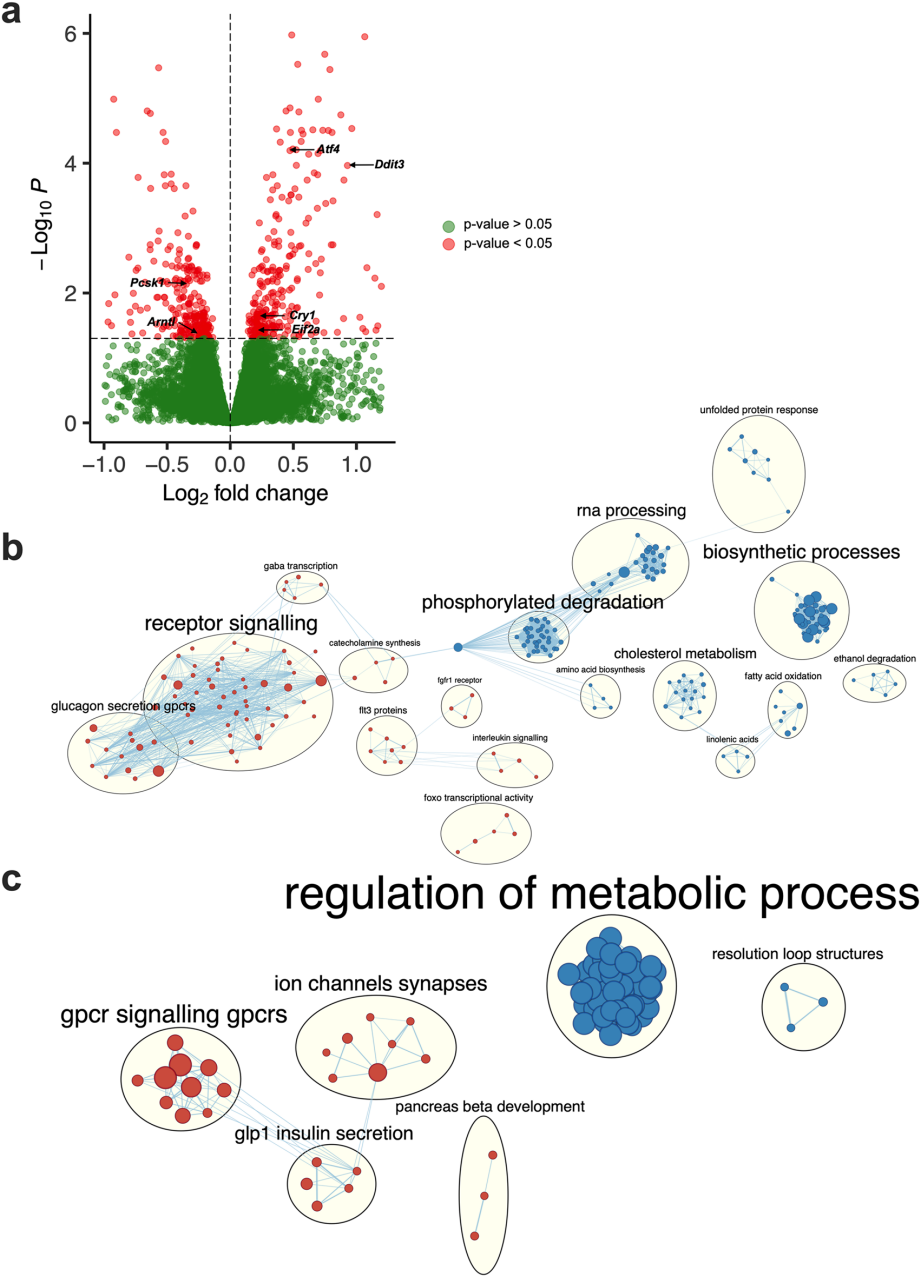
Nobiletin improves mGLUTag L cell metabolic processes following palmitate-induced disruption. (**a**) Volcano plot of the transcriptome at the peak (8 h) time point of GLP-1 secretion in synchronized palmitate-treated as compared to vehicle (control) mGLUTag L cells. (**b**, **c**) Network analysis comparing pathway enrichment between transcriptomes of (**b**) vehicle- and palmitate-treated, and (**c**) palmitate- and palmitate + nobiletin-treated mGLUTag L cells at the peak (8 h) GLP-1 secretory time point. Orange dots indicate pathways enriched to the vehicle (**b**) or palmitate (**c**) conditions; blue dots indicate pathways enriched to the palmitate- (**b**) or palmitate + NOB (**c**) treatment, with the size of the dot indicating the number of genes contained in the pathway. n = 3–4.

To provide mechanistic insight into how palmitate and nobiletin affect GLP-1 secretion, RNA-sequencing was conducted on synchronized mGLUTag L cells at the established peak (8 h) GLP-1 secretory time point (Fig. 1a and^20,22,28^). Consistent with the previously established deleterious effects of palmitate on the L cell circadian clock^27,28^, palmitate significantly (p < 0.05) suppressed expression of *Arntl* while increasing expression of *Cry1* as compared to normal L cells (Fig. 2a). However, although previous studies have shown that palmitate induces ER stress^28,36^ and impairs both mitochondrial function and ATP production^28^ in mGLUTag L cells, the entirety of its effects are unknown. Pathway analysis of the transcriptome of palmitate-treated mGLUTag L cells revealed an upregulation of ER stress and unfolded protein response related pathways (Fig. 2b; Supplementary Fig. 1a,b), including increased expression of the ER stress markers *Ddit3*, *Atf4*, and *Eif2a* (Fig. 2a). Furthermore, consistent with previous work^36^, palmitate decreased the expression of *Pcsk1*, an enzyme required for GCG processing in the L cell^37^ (Fig. 2a), paralleling the observed decrease in total GLP-1 levels. Moreover, whereas control cells exhibited increased expression of pathways related to ‘signalling’ and ‘secretion’, there was an enrichment in pathways related to fatty acid processing in palmitate-exposed cells (Fig. 2b). These data support the observations of a robust GLP-1 secretory response in response to GIP in vehicle-treated cells which was impaired upon exposure to palmitate. Furthermore, the palmitate-induced dysregulation in L cell function, largely involving induction of ER stress-related pathways, may explain the increased release of GLP-1 under basal conditions.

To understand the effects of nobiletin on L cell function, the transcriptome of nobiletin-treated mGLUTag L cells with and without palmitate exposure was next analyzed. Although nobiletin did not enhance *Arntl* expression in normal mGLUTag L cells, it did alter the expression of multiple other clock genes (Supplementary Fig. 2a). Consistent with the lack of effect of nobiletin on GLP-1 secretion in normal, vehicle-treated cells, only small differences were detected in the transcriptomic analysis. Pathways related to ‘receptor activation’ were elevated in vehicle conditions, while nobiletin increased expression of ‘metabolic processes’ (Supplementary Fig. 2b), consistent with its known role as a metabolic activator^31^. However, these beneficial effects of nobiletin were magnified upon exposure of the mGLUTag L cells to palmitate, with nobiletin profoundly increasing pathways related to ‘metabolic regulation’ that have been well-established to be key determinants of GLP-1 secretion^20,22^ (Fig. 2c; Supplementary Fig. 3a). Furthermore, nobiletin altered expression of numerous clock and exocytotic genes in the presence of palmitate, the majority of which were suppressed (Supplementary Fig. 3b) in parallel with the nobiletin-induced decrease in basal GLP-1 secretion. Concomitantly, relative to the palmitate + nobiletin-treated cells, palmitate alone induced an enrichment in pathways related to GLP-1 secretion (Fig. 2c) which, taken together with the increased ER stress in these cells (Fig. 2b), provides further evidence for palmitate-induced dysregulation in L cell function and associated increase in basal GLP-1 release. In combination, RNA-sequencing analysis of the mGLUTag L cells revealed a disruptive role of palmitate (mainly through induction of cellular stress) which is reversed by nobiletin-induced activation of metabolic pathways, in association with normalization of both the basal and GIP-stimulated peak of GLP-1 secretion.

### Nobiletin improves metabolic parameters in high-fat diet fed mice

**Figure 3:**
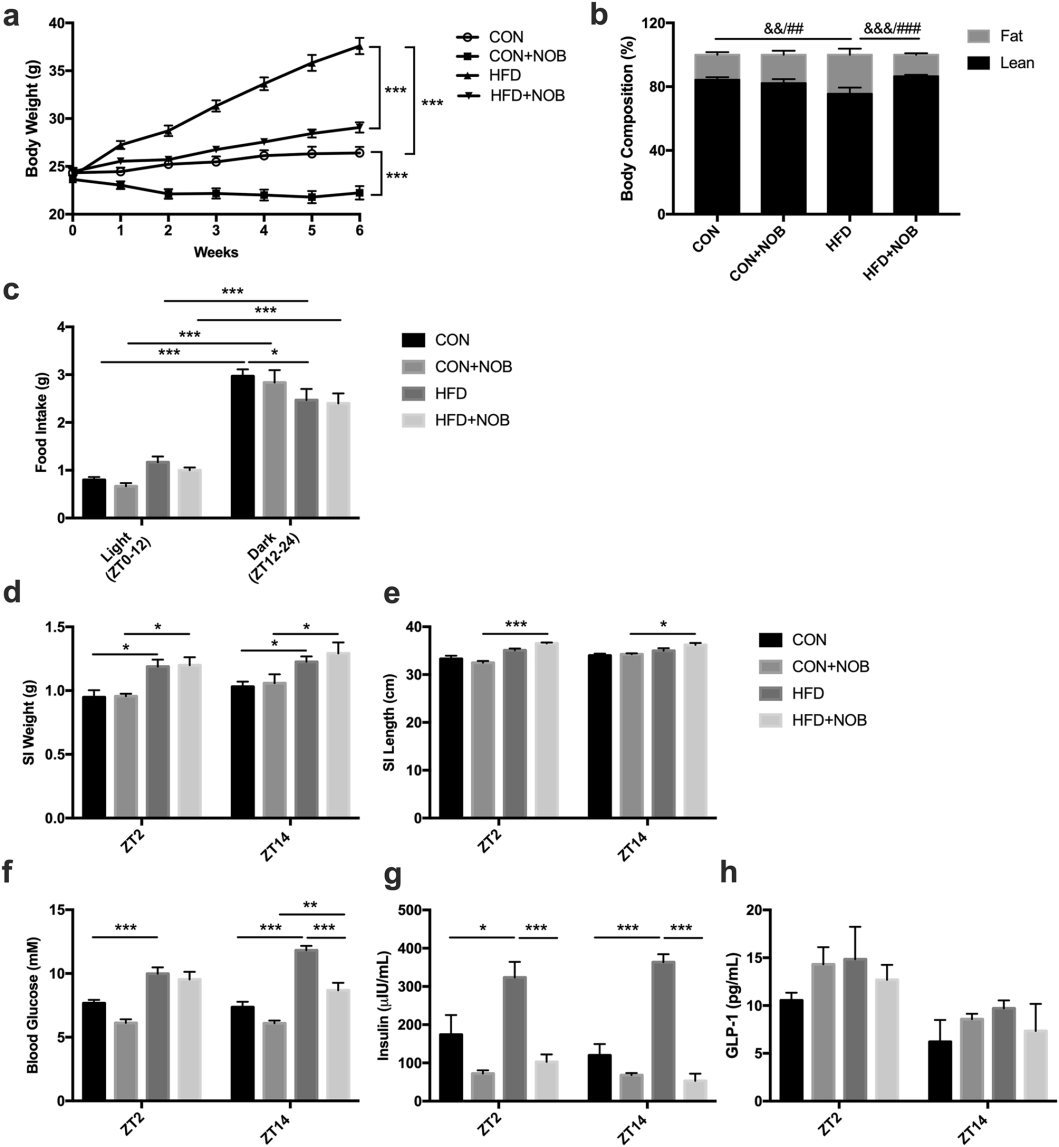
Nobiletin improves metabolic parameters following HFD-feeding in mice. (**a**) Body weight, (**b**) body composition, and (**c**) 12-h light/12-h dark food intake in control (CON)- and high-fat diet (HFD)-fed mice with and without nobiletin (NOB) supplementation. Small intestinal (**d**) weight and (**e**) length, and 4-h fasting (**f**) blood glucose, (**g**) plasma insulin, and (**h**) plasma GLP-1 at ZT14 in CON- and HFD-fed mice with and without NOB supplementation. n = 6; *p < 0.05 **p < 0.01 ***p < 0.001; ^##^p < 0.01 ^###^p < 0.001 comparing %fat composition and ^&&^p < 0.01 ^&&&^p < 0.001 comparing %lean composition.

To complement the in vitro studies, a C57Bl6/J mouse model of HFD-induced obesity, fed a CON or HFD with and without nobiletin supplementation was interrogated. As expected, HFD-fed animals had increased body weight (p < 0.001) with reduced lean mass (p < 0.01) and increased body fat (p < 0.01) as compared to CON mice (Fig. 3a,b). Consistent with previous studies^31,38^, NOB treatment reduced body weight gain in both CON- and HFD-fed animals (p < 0.001), while also improving body composition by increasing lean (p < 0.001) and decreasing fat mass (p < 0.001) in the HFD mice (Fig. 3a,b). The daily food intake pattern, an established driver of the GLP-1 secretory rhythm^19^, demonstrated that all groups of mice consumed more food during the dark/active period (p < 0.001) as compared to the light/inactive period (Fig. 3c). Furthermore, in line with previous evidence regarding HFD-feeding being a disruptor of feeding rhythms^39^, HFD-fed mice had decreased food intake during the dark/active period as compared to CON animals (p < 0.05) (Fig. 3c). However, in parallel with previous work^31^, nobiletin did not significantly affect food intake patterns either CON or HFD-fed mice (Fig. 3c). This is consistent with previous studies demonstrating that the positive metabolic benefits of nobiletin are associated with increased energy expenditure rather than altered energy intake^31^.

Given the recently reported ability of nobiletin to affect the small intestine (SI)^35^, SI weight and length were assessed at the onset of the fasting (ZT2) and feeding (ZT14) periods. Although no significant time-dependent differences were observed in SI weight or length in CON mice, HFD-feeding increased SI weight at both ZT2 and ZT14 (p < 0.05) irrespective of the presence of nobiletin, while also increasing SI length at both time points in the nobiletin-treated HFD animals (Fig. 3d,e). Finally, to establish the basal metabolic profile of the mouse model, 4-h fasting blood glucose, insulin, and GLP-1 were measured at ZT2 and ZT14. Fasting glycemia and insulinemia were elevated at both time points in HFD-fed mice as compared to CON animals (p < 0.001) (Fig. 3f,g), likely due to insulin resistance conferred by the increased body weight and adiposity. Consistent with previous work^31^, nobiletin improved fasting blood glucose (p < 0.01 at ZT14) as well as insulin at both time points (p < 0.001) in HFD-fed mice, while not having any profound effects in CON animals (Fig. 3f,g). Fasting GLP-1 levels did not display any significant time-, diet-, or nobiletin treatment-dependent differences (Fig. 3h).

### Nobiletin restores oral-glucose-stimulated GLP-1 secretion to more physiologic levels following HFD-induced disruption

**Figure 4:**
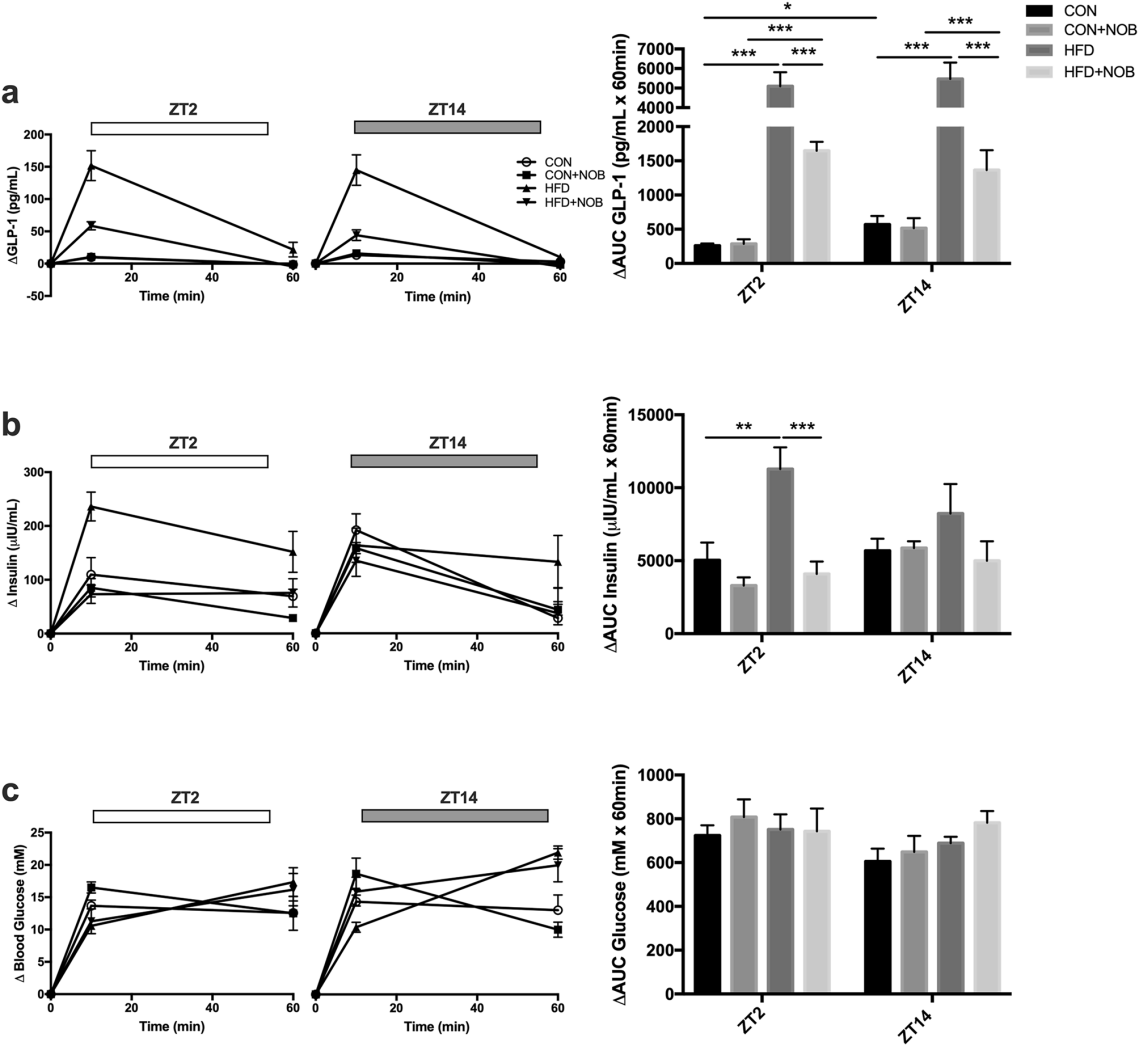
Nobiletin reduces GLP-1 secretion to more physiological levels following HFD-induced disruption. Individual responses at ZT2 and ZT14 as well as the corresponding delta area-under-the-cure (ΔAUC) for (**a**) GLP-1, (**b**) insulin, and (**c**) glucose following an OGTT in control (CON)- and high-fat diet (HFD)-fed mice with and without nobiletin (NOB) supplementation. n = 6; *p < 0.05 **p < 0.01 ***p < 0.001.

To assess the role of nobiletin in modulating GLP-1 secretion under both normal and obesogenic conditions in vivo, oral glucose tolerance tests (OGTTs) were conducted at the established trough (ZT2) and peak (ZT14) of GLP-1 secretion^20–22^. Importantly, given the differences in adiposity observed between the different groups of mice, the oral glucose load was based upon the lean mass of the animals^40^. In confirmation of previous studies^20–22^, glucose-induced GLP-1 release was greater (p < 0.05) at ZT14 as compared to ZT2 in CON animals (Fig. 4a). HFD-feeding ablated the normal secretory rhythm and resulted in a massive elevation (p < 0.001) of GLP-1 secretion at both time points (Fig. 4a), as recently reported following feeding of a high-fat/high-sucrose WD to mice^21^. Nobiletin supplementation in CON mice did not affect oral glucose-stimulated GLP-1 secretion (Fig. 4a), consistent with the notion that nobiletin does not affect metabolic homeostasis in normal mice^31^. However, interestingly, HFD + NOB mice displayed decreased GLP-1 levels at both ZT2 and ZT14 as compared to HFD alone (p < 0.001), resulting in more physiologic levels, although the normal time-dependent rhythmic secretory pattern was not re-established (Fig. 4a). In parallel with the changes observed in the GLP-1 secretory phenotype, insulin release was profoundly elevated in HFD-mice (p < 0.01 at ZT2), and this was prevented (p < 0.001) by nobiletin (Fig. 4b). The increased insulin release observed in the HFD condition is likely a compensatory mechanism for the HFD-induced insulin resistance and is responsible for the maintenance of normoglycemia in these animals which, despite several trends to changes (p = NS), were independent of time, diet, and nobiletin treatment (Fig. 4c).

### Nobiletin improves L cell metabolic processes following HFD-induced disruption

**Figure 5:**
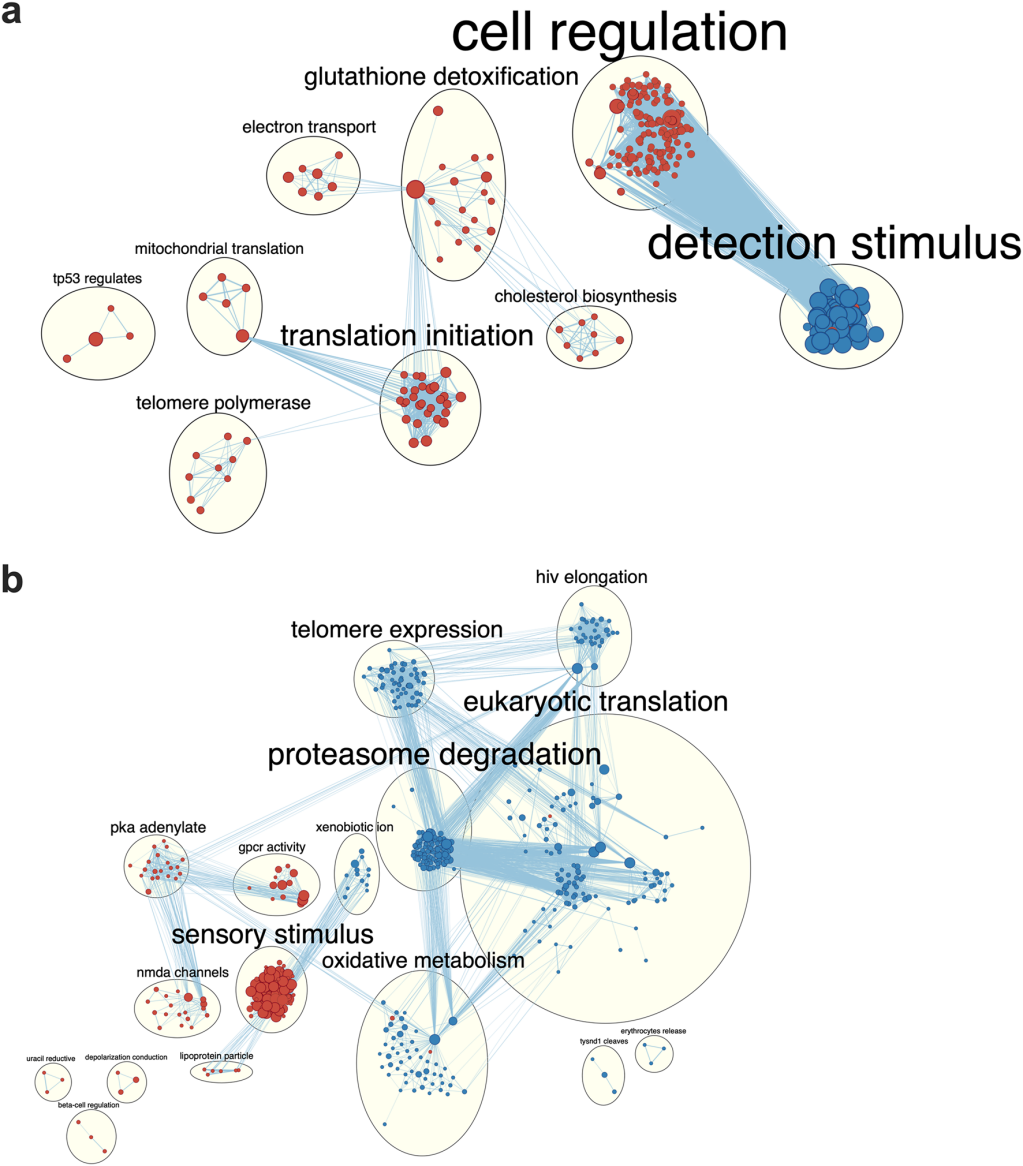
Nobiletin activates metabolic pathways in primary L cells in HFD-fed mice. Network analysis comparing pathway enrichment between transcriptomes of primary L cells isolated from (**a**) control (CON)- and high-fat diet (HFD) fed-, as well as (**b**) HFD and HFD + nobiletin (NOB) *Gcg-Venus* mice at ZT14. Orange dots indicate pathways enriched to the control (**a**) and HFD (**b**) conditions; blue dots indicate pathways enriched to the HFD (**a**) and HFD + NOB (**b**) treatments, with the size of the dot indicating the number of genes contained in the pathway. n = 3.

To complement the in vitro transcriptomic analysis, and to provide mechanistic insight into how HFD and nobiletin affect GLP-1 secretion, RNA-sequencing was performed on primary colonic L cells isolated from *Gcg-Venus* mice at the established peak time point (ZT14) of GLP-1 secretion (Fig. 4a and^20–22^). Control L cells exhibited an enrichment in pathways related to mitochondrial function as compared to L cells isolated from HFD-fed animals (Fig. 5a; Supplementary Fig. 4a). These data are in-line with a previously reported transcriptomic analysis of isolated L cells from WD-fed mice, which showed diminished mitochondrial gene expression compared to chow-fed controls^21^. Interestingly, L cells isolated from HFD-fed mice demonstrated an increase in pathways related to perception and transduction of cellular stimuli, which have been previously associated with increased GLP-1 secretion^21^ (Fig. 5a; Supplementary Fig. 4b) and is in keeping with the markedly enhanced GLP-1 secretory responses in these mice (Fig. 4a).

Consistent with the lack of an effect of nobiletin on GLP-1 secretion under CON dietary conditions, there were no profound changes at the transcriptomic level in L cells from CON vs. CON + NOB animals (Supplementary Fig. 5a). However, nobiletin induced marked changes in the L cell transcriptome in the HFD mice. Consistent with the massively elevated GLP-1 levels following HFD feeding, HFD-exposed L cells exhibited increased expression of pathways related to cellular stimulation as compared to HFD + NOB L cells (Fig. 5b). In contrast, nobiletin supplementation in HFD conditions increased L cell ‘oxidative metabolism’ pathways (Fig. 5b; Supplementary Fig. 5b), paralleling its effects on the mGLUTag L cells in vitro.

### Nobiletin improves microbial composition following HFD-induced disruption

**Figure 6:**
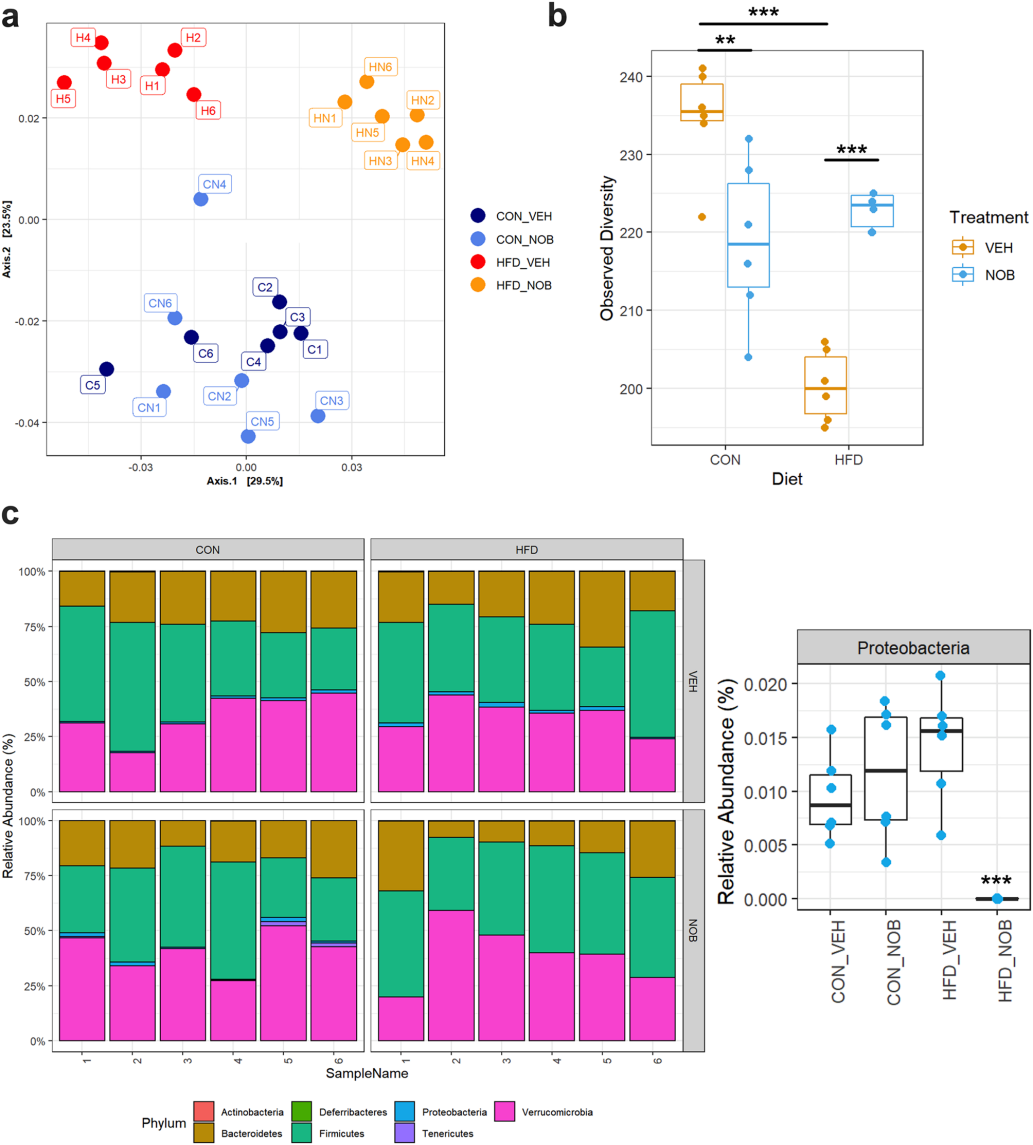
Nobiletin improves the gut microbiome in HFD-fed mice. (**a**) Principal component analysis, (**b**) species diversity, and (**c**) relative abundance of phyla, including a plot for Proteobacteria, of the colonic microbiome at ZT14 from control (CON) and high-fat diet (HFD)-fed mice without (vehicle; VEH) or with nobiletin (NOB) supplementation. n = 6, with each dot and each bar showing the data for a single animal; **p < 0.01 ***p < 0.001.

Finally, given recent work demonstrating the necessity for a normal microbiome in establishing circadian GLP-1 release^21^, we conducted 16S rRNA gene sequencing of colonic feces. As expected, HFD-feeding decreased the overall diversity of the microbial community (p < 0.001) (Fig. 6a,b). While nobiletin supplementation in CON animals did not cause significant changes in the microbiome, it did result in a decrease of microbial diversity (p < 0.01) (Fig. 6a,b). In contrast, in HFD-fed mice, nobiletin shifted the microbial composition away from the HFD phenotype as well as markedly increasing the diversity (p < 0.001) (Fig. 6a,b). Finally, although no profound changes were observed in the major phyla between CON and HFD conditions (Fig. 6c), the relative abundance of select families (i.e. *Bacteroidaceae*, *Clostridaceae_1*) was altered by HFD-feeding (Supplementary Fig. 6). Importantly, nobiletin supplementation in HFD-fed mice dramatically (p < 0.001) reduced the relative abundance of the detrimental Proteobacteria phyla (Fig. 6c). Together, these data indicate that nobiletin administration reverses many of the negative effects of HFD-feeding on the gut microbiota, in association with a partial restoration of the GLP-1 secretory phenotype.

## Discussion

The intestinal L cell has been shown to express a functional cell-autonomous circadian clock, as evidenced by rhythms in core clock gene expression and GLP-1 secretion, in vitro and in vivo^19–21^. Furthermore, studies in both whole-body and L cell *Arntl* knockout mice have identified the circadian clock to be essential for time-dependent GLP-1 release^20,22^. However, the physiologically-relevant circadian disruptors, obesogenic feeding and the saturated fatty acid palmitate, have been demonstrated to disrupt the normal GLP-1 secretory rhythm^27,28^. Herein, we demonstrate that the small molecule, nobiletin, restores the normal GLP-1 secretory response following exposure of L cells to palmitate in vitro, largely as a result of reducing basal secretion to normal levels. Mechanistically, palmitate-induced L cell dysfunction was characterized by ER stress, with nobiletin administration resulting in activation of metabolic processes and normalization of the GLP-1 secretory response. In vivo, nobiletin ameliorated the GLP-1 secretory phenotype by returning oral glucose-induced GLP-1 release to more physiological levels following disruption by HFD-feeding in vivo. Transcriptomic analysis revealed an overstimulated L cell secretory phenotype following exposure to HFD-feeding, with nobiletin increasing cellular metabolic pathways to improve L cell function.

Previous studies in synchronized mGLUTag L cells have demonstrated disruptive effects of palmitate on the amplitude of *Arntl* and *Per2* clock gene expression^27^. Specifically, at the peak GLP-1 secretory time point (8 h post-synchronization), *Arntl* mRNA expression was dampened by palmitate^27,28^, a finding that was confirmed by the transcriptomic data in the present study. Somewhat surprisingly, nobiletin, an established RORα-dependent enhancer of the circadian amplitude of *Arntl*^31,33,34^, did not increase *Arntl* expression at the 8-h time point, either alone or in the presence of the dampening effects of palmitate and, indeed, changes in *Rora* were not detected in any of the L cell transcriptomic analyses conducted herein, although disruption in many other clock genes were detected. It is also important to note that, in select contexts the actions of nobiletin were found to be clock-independent^33,34^, which may account for the present observations on the L cell. This finding may also be explained by the single sampling time point used in the present study, which precludes detection of any changes that occurred at other time points in the circadian cycle. Moreover, studies using nobiletin in clock-disrupted L cell models may provide insight into whether the nobiletin-induced activation of cellular metabolic and oxidative pathways observed herein requires a functional circadian clock.

Previous transcriptomic and proteomic data identified that pathways related to ‘nutrient sensing’ and ‘cellular metabolism’ are enriched to the peak timepoint of GLP-1 release^20–22^. At the molecular level, palmitate suppresses L cell BMAL1 protein expression in the mGLUTag cells, which impairs mitochondrial function and ATP production and, ultimately, results in decreased stimulated GLP-1 secretion^28^. Furthermore, palmitate has also been shown to disrupt normal L cell physiology, irrespective of time, through the induction ER stress^28,36^. As normal L cells had an enrichment in pathways related to ‘signalling’ and ‘secretion’, this provides a mechanistic basis for the observed impairment in the GLP-1 response to GIP in palmitate-exposed L cells. Surprisingly, analysis of the mGLUTag L cell transcriptome following palmitate exposure did not identify dysregulation in pathways related to ‘mitochondrial function’, which we had previously identified to be disrupted by palmitate^28^. However, previous work focused on functional cellular changes through direct assessment of mitochondrial activity^28^, which may not be reflected at the mRNA level as assessed in the present study. However, transcriptomic analysis did confirm the disruptive effects of palmitate treatment on the L cell through induction of a profound increase in ER stress-related pathways. The abrogation of these changes with nobiletin co-administration, in the setting of upregulated ‘metabolic processes’ was thus established to be a key mechanism underlying the normalization of GLP-1 release, in palmitate-disrupted L cells.

Importantly, previous work determined that expression of genes involved in SNARE-mediated exocytosis is enriched to the peak time point of GLP-1 release^20–22^. In the present study, nobiletin reduced basal GLP-1 levels in the presence of palmitate through alterations in expression of the SNARE proteins, decreasing *Vamp2*, a SNARE protein that is essential for basal and stimulated GLP-1 release^41^, as well as *Snap25* and *Syn7*, a calcium-dependent SNARE accessory protein that regulates GLP-1 release^42^. Concomitantly, nobiletin increased expression of *Stx1a*, a SNARE protein important for stimulated but not basal GLP-1 secretion^43^. Although no differences in expression of genes related to SNARE-mediated exocytosis were observed in vivo, this discrepancy which may be explained by the inherent difference between a controlled in vitro setting and the more complex nature of the primary L cell which receives input from a variety of sources in the body, including the microbiome.

Previous work focusing on the effects of obesogenic feeding on GLP-1 release has produced conflicting evidence based on the models used. Several studies using both mice and rats have reported that HFD feeding results in impaired GLP-1 secretion^44–46^; however, these differed from the present work in terms of the animal model, dietary composition and duration of feeding and fasting times, as well as in the oral glucose load, making a direct comparison impossible. However, feeding of a high-fat/high-sucrose WD to mice results in elevated GLP-1 levels and loss of the normal secretory rhythm^21^, consistent with the results observed herein with a HFD. Following administration of an oral glucose load that was based upon lean mass^40^, nobiletin decreased the GLP-1 secretory response to more physiological levels in HFD-fed animals. At the mechanistic level, L cells exhibited impaired mitochondrial activity as a result of HFD feeding, and likely through its effect to increase ‘oxidative metabolism’ pathways, nobiletin treatment restored L cell function as reflected in more physiological levels of GLP-1 secretion. Notably, the nobiletin-induced improvement in the GLP-1 secretory phenotype was mirrored by a normalization of the response in insulin, consistent with findings that rhythmic GLP-1 release serves as a zeitgeber for the normal time-dependent patterns in insulin release^19,21,26^. These findings are also in-line with the well-established^31^ beneficial metabolic effects of nobiletin which include reductions in body weight and improvements in body composition, as well as in fasting glycemia and insulinemia in HFD-fed mice. Furthermore, given that both insulin and leptin are established stimulators of GLP-1 secretion^44,47^, the nobiletin-induced reduction in body weight would also decrease the levels of both of these hormones thereby, at least in part, returning GLP-1 to more physiological levels.

Interestingly, although palmitate suppressed the GLP-1 secretory response in vitro, stimulated GLP-1 secretion in HFD-fed mice was massively elevated. This discrepancy between the in vitro and in vivo findings is not unique to the present study, as our recent work investigating GLP-1 secretion in mice fed a WD also demonstrated an increase in GLP-1 release in vivo but normal secretion by primary intestinal cultures generated from those animals in vitro^21^. As noted above, these differences could be explained by the notion that GLP-1 secretion is subject to hormonal modulation in vivo which is not present in cell culture. However, our recent work has also positioned the gut microbiome as a key in vivo factor that is essential for both time- and diet-dependent differences in GLP-1 release^21^.

In line with the well-established literature^48^, the fecal microbiome was profoundly affected by high-fat feeding, with a markedly reduced diversity as compared to control mice. Our previous work demonstrated that the species *Akkermansia muciniphila* of the Verrucomicrobia phylum was increased at ZT14 compared to ZT2, and was both increased and arrhythmic following WD-feeding, in parallel with the time- and diet-dependent changes in GLP-1 secretory responses^21^. However, in the present study, the increased GLP-1 secretion observed following HFD feeding did not seem to be a result of an increase in *A. muciniphila*. This discrepancy may be explained by differential dietary compositions as well as the duration of the feeding period. However, the beneficial effect of nobiletin to improve the microbial community following HFD-induced disruption reported herein is consistent with previous work^38,49^. Although it has been reported that nobiletin administration increases the abundance of several beneficial species, such as *Akkermansia* and *Bacteroides*^38^, this was not replicated in the present study. Indeed, the major effect of nobiletin in the present study was the complete abrogation of Proteobacteria, a phyla associated with microbial dysbiosis^50^. Interestingly, a recent report indicates that duration of nobiletin administration may be important for its effects on the gut microbiome, with 4 weeks producing an improvement in microbial diversity, an effect that is lost by 8 weeks of treatment^49^. Therefore, the discrepancy between the literature and the data presented herein is likely a result of differences in dosage and duration of administration of nobiletin.

While the present study identified a number of mechanisms by which exposure to high fat and nobiletin in vitro and in vivo impact L cell secretory function, it is important to consider the limitations. Although our previous studies have not identified sex-differences in GLP-1 secretion^20–22^, the present study made use of only male mice, requiring future studies of female animals. Furthermore, most of the studies were conducted at a single time point which, although selected to be at the peak of the GLP-1 secretory rhythm, precludes any conclusions regarding the circadian nature of the findings. Future studies will be required to elucidate the effects of high-fat and nobiletin on L cell function and GLP-1 secretion over the course of 24-h. Notwithstanding, the present findings provide further detail regarding the effects of high-fat exposure on the L cell and clearly demonstrate the potential for nobiletin to ameliorate the resultant impairments in GLP-1 secretion. Given that GLP-1 has been determined to be an important regulator of rhythmic insulin secretion and diurnal glucose homeostasis^19,21,26^, restoring physiologic rhythms within L cells may be more beneficial than overriding them with the currently-available long-lasting GLP-1-derivative drugs. Maintenance of these physiological rhythms may also be important to the timing of GLP-1 secretagogues. The current findings on the role of the clock-enhancer nobiletin, may therefore provide insight into the development of novel therapeutic strategies targeting improved L cell function in metabolic disease.

## Methods

### In vitro studies

#### Cell culture

The male colonic murine (m) GLUTag L cell line was chosen as a model of the intestinal L cell due to similarity in regulation of GLP-1 secretion to primary L cells, including a cell-autonomous circadian clock. mGLUTag L cells were cultured and synchronized as previously described^19,20,22,27,28,51^. Briefly, cells were grown on 6-well plates in Dulbecco’s modified Eagle’s medium with 25 mmol/L glucose and 10% FBS for 48-h. Cells were then pre-treated for 12-h with vehicle or 0.5 mM palmitate (PAL; Sigma-Aldrich) made by dissolving in water at 70 °C and then adding to growth medium, as previously described^27,28^. Cells were also treated with vehicle or 20 μM NOB (R&S PharmChem: ≥ 98% pure; and Sigma-Aldrich; ≥ 97% pure; dissolved in DMSO). Cells were synchronized by incubation for 12-h in 0.5% FBS media followed by addition of 20 μM forskolin in 10% FBS media for 1-h. Media were then changed to regular growing media with the appropriate treatments for an additional 8-h to reach the established temporal peak of GLP-1 secretion^19,20,22,28^. At the 8-h time point, cells were incubated for 2-h in 0.5% FBS media containing vehicle (CON) or the known secretagogue, 10^−7^ M glucose-dependent insulinotrophic polypeptide (GIP) for GLP-1 secretion assay (n = 6) or total RNA was extracted using the RNeasy Plus Mini Kit with QIAshredder (Qiagen) for RNA-sequencing analysis (n = 3–4).

#### GLP-1 secretion assay

Peptide extraction from the media and cells was conducted by reversed-phase adsorption using C18 Sep-Pak cartridges (Waters Associates). GLP-1 levels were measured using the Total GLP-1 Radioimmunoassay kit (GLP-1 T-36HK, Millipore). GLP-1 secretion was expressed as the percent of the GLP-1 in the media over the total GLP-1 content (media + cells).

### In vivo studies

#### Animal model

5–7-week-old male C57Bl6/J mice (Jackson Laboratories) were randomly assigned into treatment groups (n = 6) and allowed to acclimate for one week. Male proglucagon (*Gcg*)*-Venus* C57Bl/6 J mice^52^ (n = 3) were bred at the University of Toronto and used at 5–7-weeks of age. Throughout the duration of the experiment, mice were housed in a 12-h light/12-h dark cycle at constant room temperature with ad libitum access to food and water. Mice were fed a high-fat diet (HFD; 60% calories from fat; D12492, Research Diets) for 6 weeks to achieve a state of diet-induced obesity, with a sucrose-matched regular chow diet (CON; 10% calories from fat; D12450J, Research Diets) used as control. A subset of animals in both diet groups was supplemented, as previously described, with 0.3% w/w nobiletin (R&S PharmChem)^32,35^. In brief, dietary pellets were mashed together with nobiletin powder and reformed into pellets. To avoid taste aversion, the nobiletin dose was gradually increased over the course of the first week^32,35^. All mice were weighed weekly, body composition (lean and fat tissue) was determined by Dexa Scan following the 6-week feeding period, and total food intake was measured for the 12-h light and dark periods. All animal studies were approved by the Animal Care Committee at the University of Toronto, all methods were in accordance with the relevant guidelines and regulations of the Canadian Council on Animal Care, and our reporting follows the recommendations of the ARRIVE Guidelines.

#### Oral Glucose Tolerance Tests (OGTTs)

OGTTs (5 g glucose/kg lean tissue mass) were conducted on 4-h fasted mice at zeitgeber (ZT; hours after lights-on) 2 and ZT14 (the established trough and peak of GLP-1 secretion, respectively)^20–22^. A basal blood sample was collected from the tail vein prior to the glucose gavage at t = 0 min, with additional samples taken at t = 10 and 60 min. Blood glucose was determined using a OneTouch meter (LifeScan), and plasma total GLP-1 and insulin were analyzed by MesoScale Discovery Assay.

#### Primary L cell isolation and transcriptomic analysis

Primary colonic L cells from *Gcg-Venus* mice were collected at ZT14 by fluorescence-activated cell sorting using a BD FACS Melody cell sorter (BD Bioscience) at the University of Toronto Flow Facility, as previously described^21^. Briefly, DAPI-staining as well as side- and forward-scatter were used to remove non-Venus positive and dead cells. Venus-positive cells were isolated into LoBind tubes (Eppendorf) with RLT-Buffer (Qiagen). Total RNA was extracted using the RNeasyPlus Micro Kit (Qiagen) with gDNA removal. Samples were delivered to the Donnelly Sequencing Centre for library preparation and sequencing.

#### RNA-sequencing

As previously described^21,22^, RNA extracted from mGLUTag L cells as well as that from primary L cells was sequenced using 150 base paired-end reads. Sequencing data were then mapped using kallisto^53^ and count tables were generated using tximport^54^. Data were normalized using Edge R^55^, with genes of low expression removed. Differential expression analyses were performed using limma^56^ (Bioconductor) with an adjusted p-value of 0.01. Gene set enrichment analysis was conducted using the limma function camera using the Bader Lab gene set resource (http://download.baderlab.org/EM_Genesets). Network analyses were generated using the EnrichmentMap^57,58^ plug-in in Cystoscope, where nodes represent up- or down- regulated pathways. Edges connecting nodes were established through an overlap score based on the number of shared genes between pathways. Clusters of functionally related pathways were then grouped and labelled using AutoAnnotate.

#### Microbiome analysis

As previously described^21,22^, colonic fecal samples were collected and delivered to the Centre for the Analysis of Genome Evolution & Function at the University of Toronto for DNA extraction, 16S rRNA gene sequencing, and analysis. Briefly, colonic fecal DNA was extracted using the ZymoBIOMICS DNA miniprep kit (ZymoBIOMICS), the V4 hypervariable region of the 16S rRNA gene was amplified, and the sequencing data analysis was conducted using the UNOISE pipeline, available through USEARCH.

### Statistical analyses

Delta area-under the curve was determined using the trapezoidal rule after subtracting fasting levels. Statistical analyses (with the exception of the RNA-sequencing data, as noted above) were conducted using GraphPad Prism with all data being expressed as mean ± SEM. ANOVA (2- or 3-way) followed by Tukey’s post-hoc analysis, as appropriate, was used to determine significance, which was set at p < 0.05.

## Supplementary Information

Supplementary Figures.

## Data Availability

The datasets generated and/or analysed during the current study are available in the ArrayExpress repository at https://www.ebi.ac.uk/arrayexpress/experiments/E-MTAB-11539 for the mGLUTag L cells, https://www.ebi.ac.uk/arrayexpress/experiments/E-MTAB-11538 for the primary L cells, and https://www.ebi.ac.uk/arrayexpress/experiments/E-MTAB-11551 for the 16S rRNA gene sequencing.

## Abbreviations

GIP: Glucose-dependent insulinotrophic polypeptide
GLP-1: Glucagon-like peptide-1
HFD: High-fat diet
T2D: Type 2 diabetes
WD: Western diet
ZT: Zeitgeber
