# Supplementary material for: Nobiletin ameliorates high fat-induced disruptions in rhythmic glucagon-like peptide-1 secretion
Source: Sci Rep. 2022 May 4;12:7271. doi: 10.1038/s41598-022-11223-7 (PMC9068808; doi:10.1038/s41598-022-11223-7)

Nobiletin ameliorates high fat-induced disruptions in rhythmic glucagon-like peptide-1 secretion

Alexandre Martchenko[1], Andrew D. Biancolin[1], Sarah E. Martchenko[1], Patricia L. Brubaker[1,2]

Departments of [1]Physiology, and [2]Medicine, University of Toronto,

Toronto, ON Canada

Supplementary Figure Legends

Supplementary Figure 1: Representative (**a**) ER stress and (**b**) unfolded protein response gene sets comparing palmitate-treated to control (vehicle) mGLUTag L cells at the peak (8 hour) GLP-1 secretory time point.

Supplementary Figure 2: (**a**) Volcano plot and (**b**) network analysis comparing pathway enrichment of the transcriptomes of nobiletin to vehicle-treated mGLUTag L cells at the peak (8 hour) GLP-1 secretory time point. Orange dots indicate pathways enriched to the vehicle condition; blue dots indicate pathways enriched to the nobiletin-treated condition, with the size of the dot indicating the number of genes contained in the pathway. n=3-4

Supplementary Figure 3: (**a**) Representative cellular metabolic processes gene set and (**b**) volcano plot comparing palmitate+nobiletin- to palmitate-treated mGLUTag L cells at the peak (8 hour) GLP-1 secretory time point. n=3-4

Supplementary Figure 4: Representative (**a**) mitochondrial function and (**b**) cellular stimulation gene sets comparing transcriptomes of primary L cells isolated from HFD- to CON-mice at ZT14. n=3-4

Supplementary Figure 5: (**a**) Network analysis comparing pathway enrichment between transcriptomes of primary L cells isolated from CON+nobiletin to CON mice at ZT14. Orange

dots indicate pathways enriched to the CON condition; blue dots indicate pathways enriched to the CON+ nobiletin condition, with the size of the dot indicating the number of genes contained in the pathway. (**b**) Representative 'oxidative metabolism' gene sets comparing transcriptomes of primary L cells isolated from HFD+ nobiletin to HFD-mice at ZT14. n=3-4

Supplementary Figure 6: Relative abundance of families of the colonic microbiome at ZT14 from control (CON) and high-fat diet (HFD)-fed mice without (vehicle; VEH) and nobiletin (NOB) supplementation. n=6, with bars showing the data for a single animal.

Supplementary Figure 1

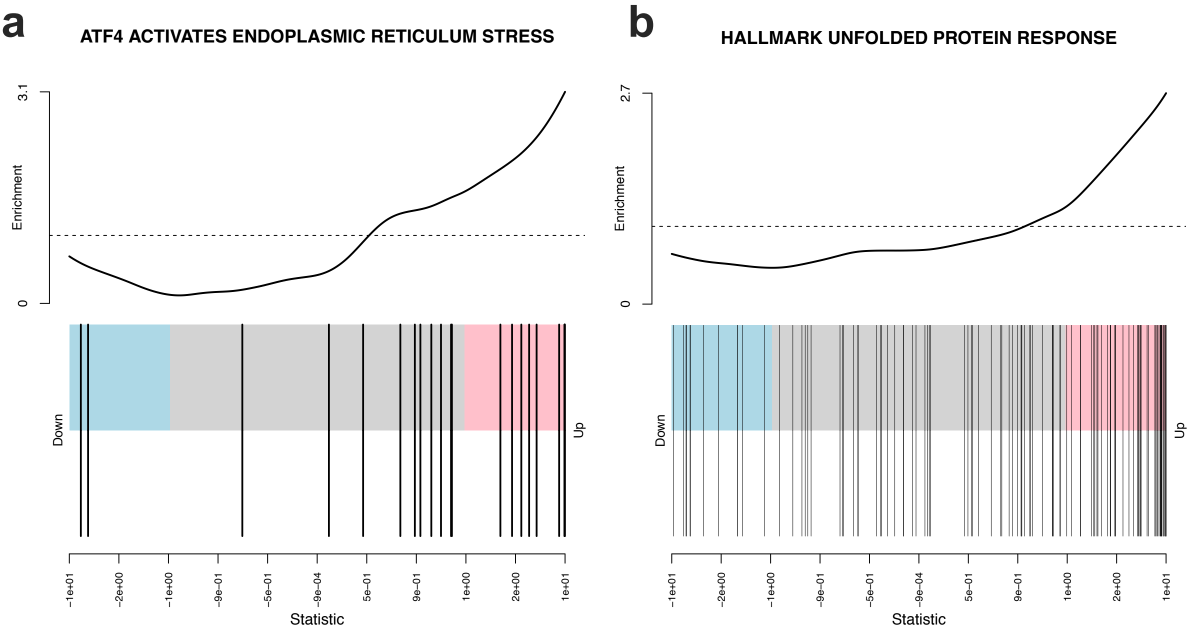

Supplementary Figure 2

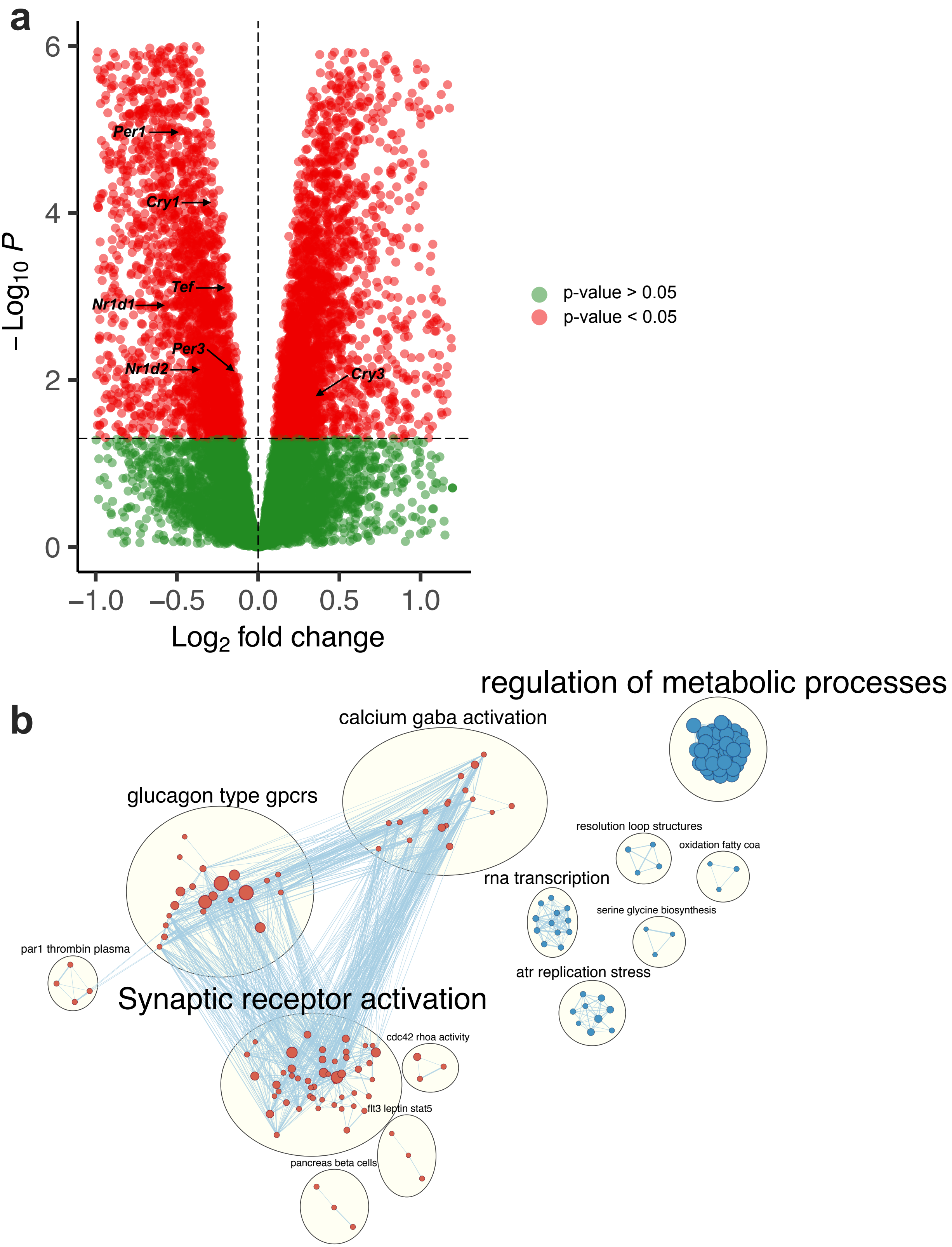

Supplementary Figure 3

a

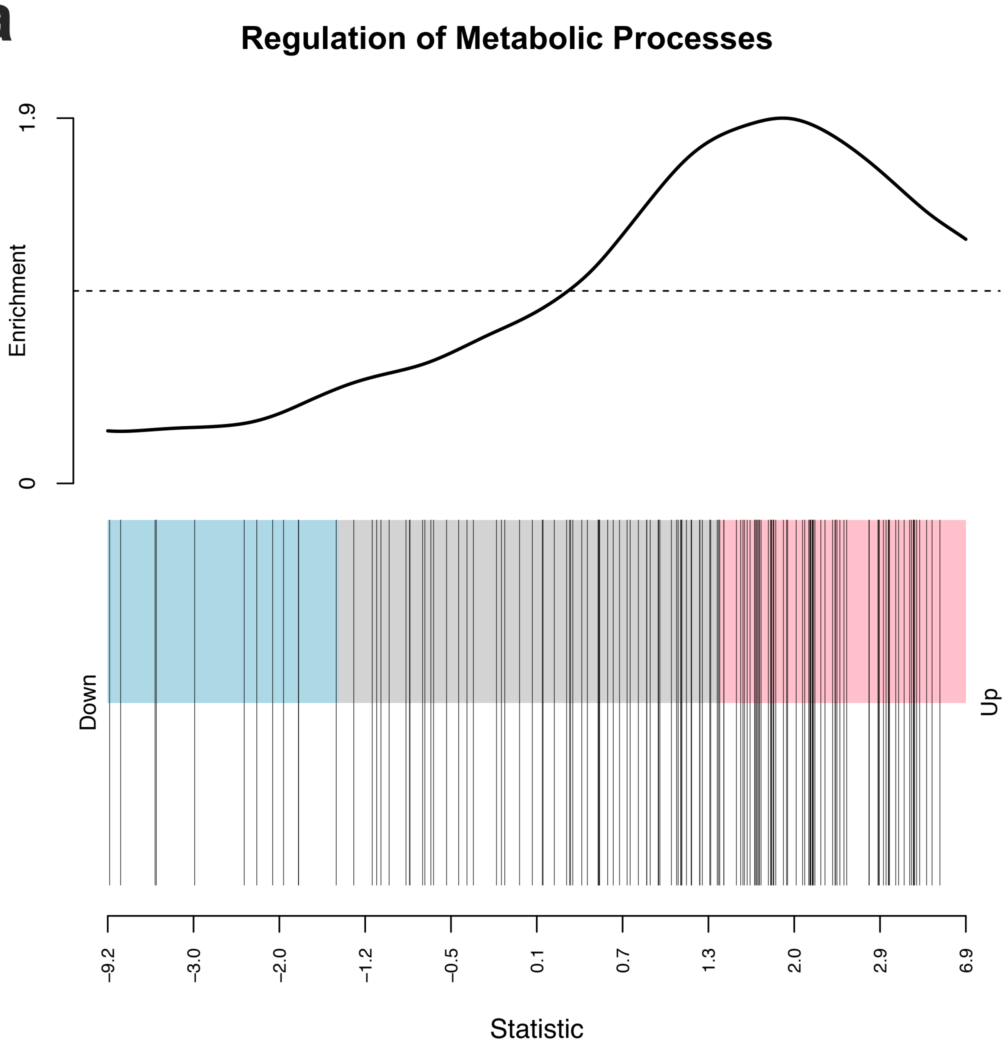

b

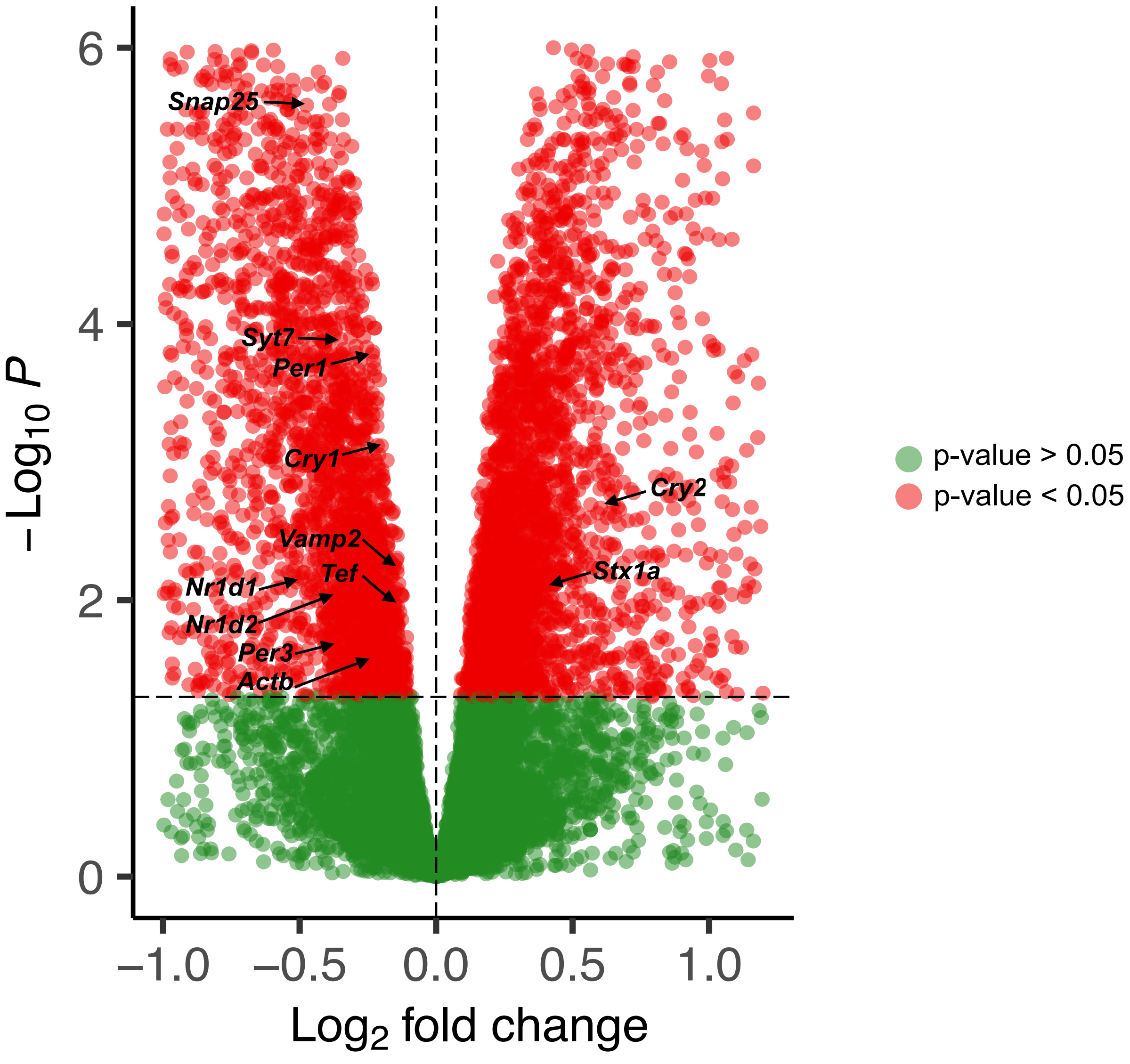

Supplementary Figure 4

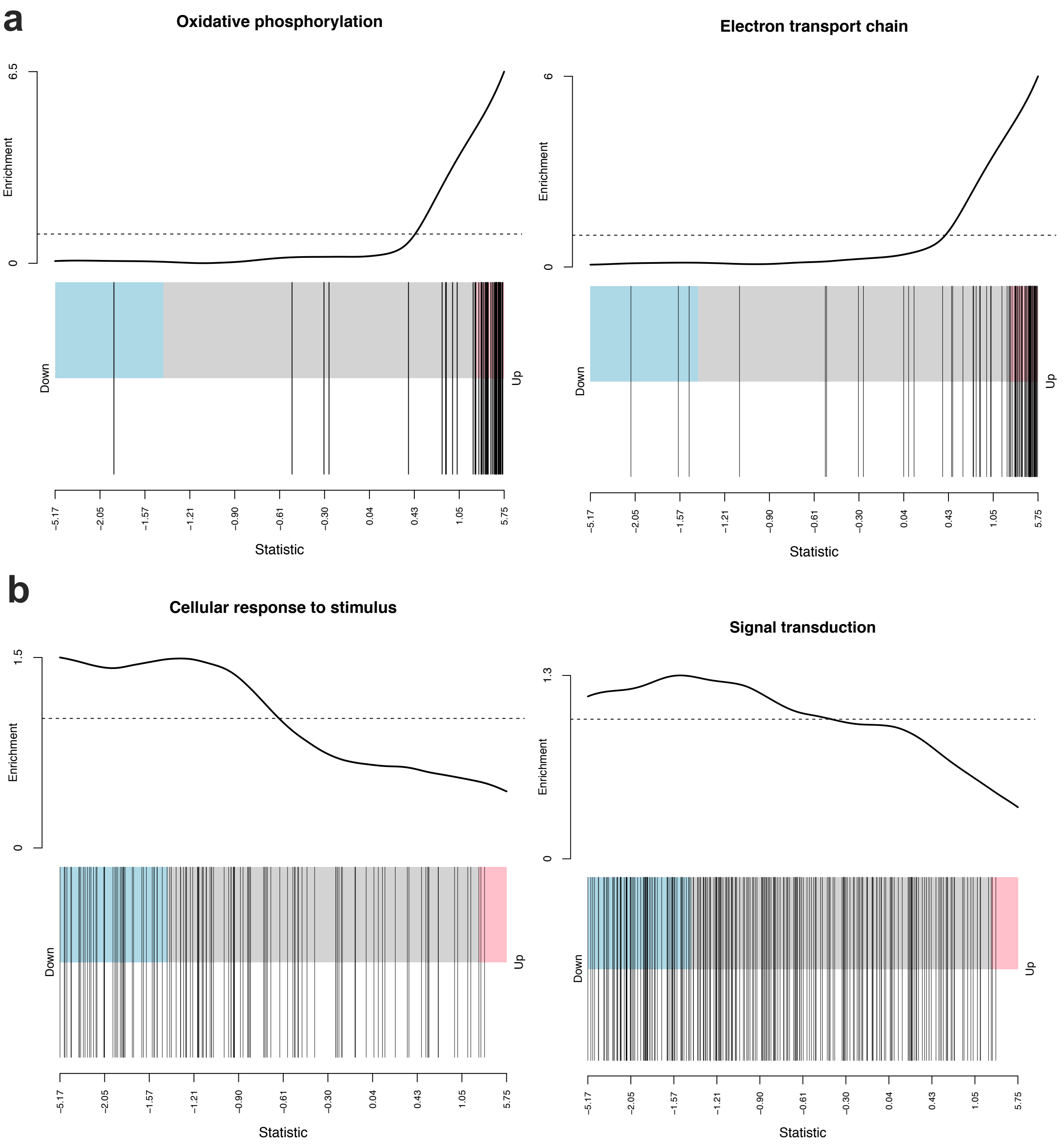

Supplementary Figure 5

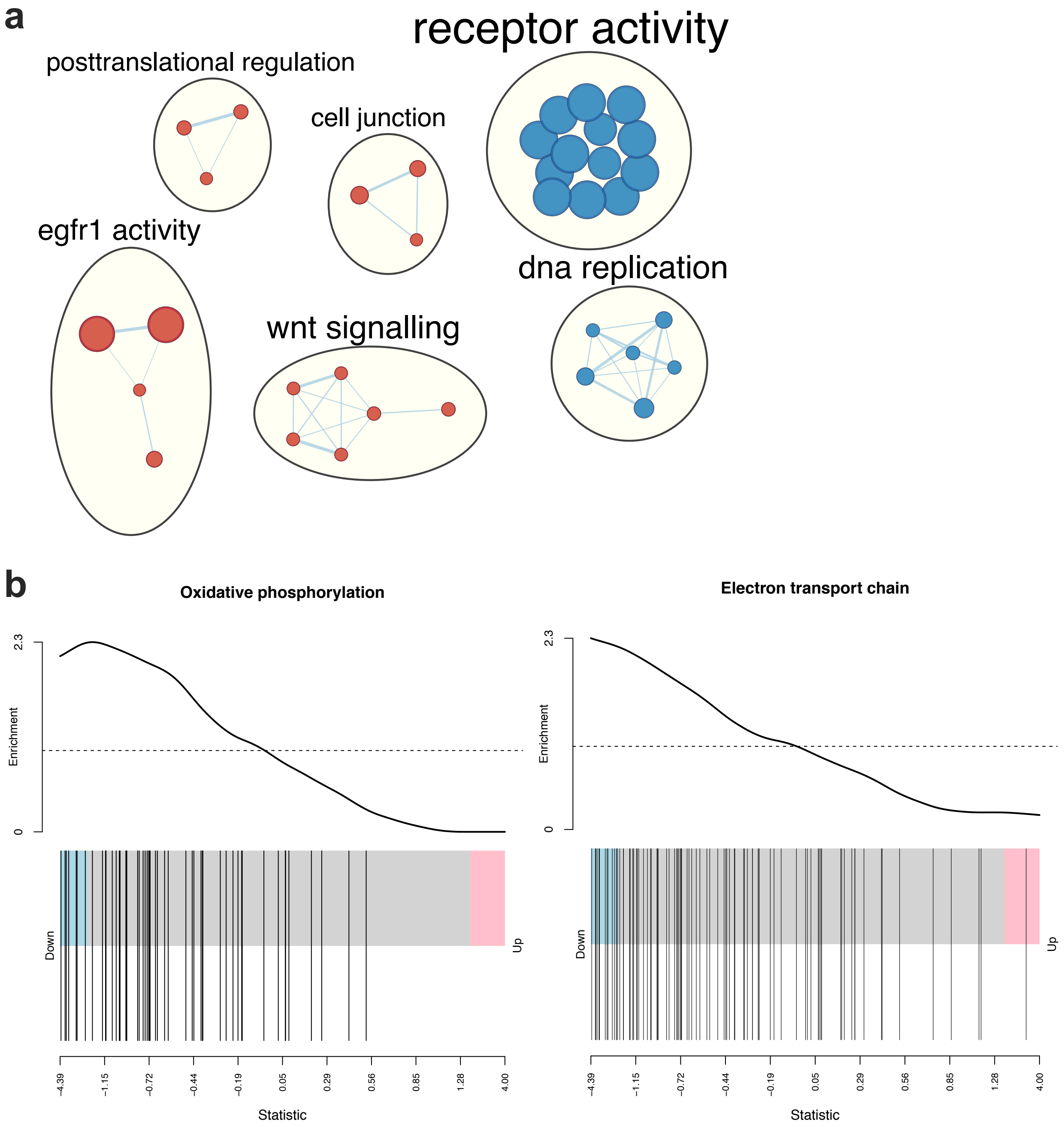

Supplementary Figure 6

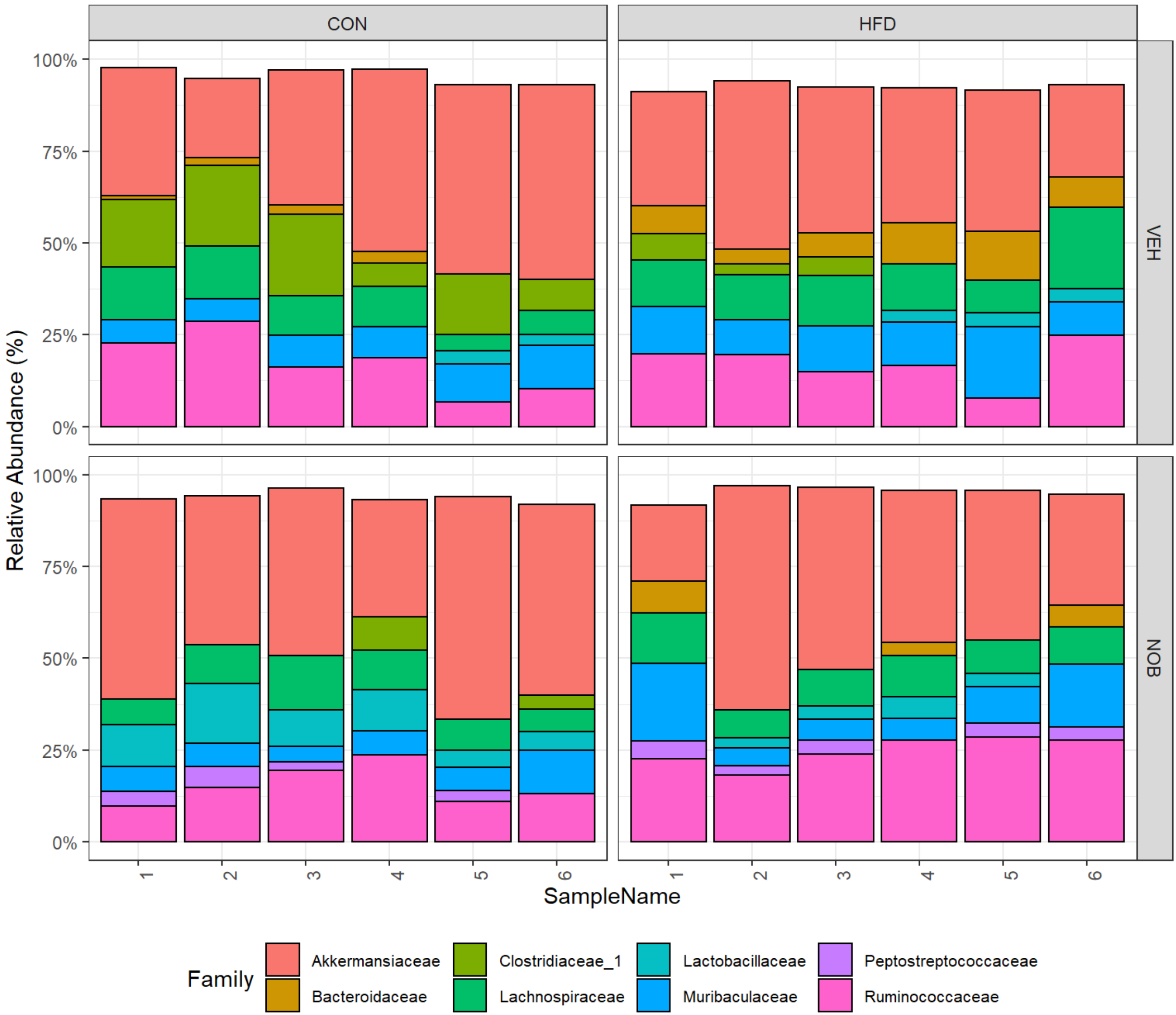

Supplement: Supplementary file 1 — Supplementary Figures. [file 41598_2022_11223_MOESM1_ESM.pdf]
